# A Texture Reconstructive Downsampling for Multi-Scale Object Detection in UAV Remote-Sensing Images

**DOI:** 10.3390/s25051569

**Published:** 2025-03-04

**Authors:** Wenhao Zheng, Bangshu Xiong, Jiujiu Chen, Qiaofeng Ou, Lei Yu

**Affiliations:** 1 The School of Information Engineering, Nanchang Hangkong University, Nanchang 330063, China; 2204081100011@stu.nchu.edu.cn (W.Z.); chenjiujiu@nchu.edu.cn (J.C.); ou.qiaofeng@nchu.edu.cn (Q.O.); yulei@nchu.edu.cn (L.Y.); 2 The Key Laboratory of Jiangxi Province for Image Processing and Pattern Recognition, Nanchang 330063, China

**Keywords:** UAV, object detection, remote sensing, downsampling, back-projection

## Abstract

Unmanned aerial vehicle (UAV) remote-sensing images present unique challenges to the object-detection task due to uneven object densities, low resolution, and drastic scale variations. Downsampling is an important component of deep networks that expands the receptive field, reduces computational overhead, and aggregates features. However, object detectors using multi-layer downsampling result in varying degrees of texture feature loss for various scales in remote-sensing images, degrading the performance of multi-scale object detection. To alleviate this problem, we propose a lightweight texture reconstructive downsampling module called TRD. TRD models part of the texture features lost as residual information during downsampling. After modeling, cascading downsampling and upsampling operators provide residual feedback to guide the reconstruction of the desired feature map for each downsampling stage. TRD structurally optimizes the feature-extraction capability of downsampling to provide sufficiently discriminative features for subsequent vision tasks. We replace the downsampling module of the existing backbone network with the TRD module and conduct a large number of experiments and ablation studies on a variety of remote-sensing image datasets. Specifically, the proposed TRD module improves 3.1% AP over the baseline on the NWPU VHR-10 dataset. On the VisDrone-DET dataset, the TRD improves 3.2% AP over the baseline with little additional cost, especially the ${\mathrm{AP}}_{S}$, ${\mathrm{AP}}_{M}$, and ${\mathrm{AP}}_{L}$ by 3.1%, 8.8%, and 13.9%, respectively. The results show that TRD enriches the feature information after downsampling and effectively improves the multi-scale object-detection accuracy of UAV remote-sensing images.

## 1. Introduction

Object detection is a fundamental task in computer vision, primarily involving label classification and bounding box localization of objects in images or videos. Detection of unmanned aerial vehicle (UAV) images has been a popular task recently. With the continuous development of deep-learning neural networks and the emergence of large-scale aerial image datasets, the detection of UAV images has already made a significant impact in the fields of traffic monitoring [1], urban security [2], and emergency rescue [3], generating considerable low-altitude economic benefits. Real-time, high-precision detection of UAV images can provide sustainable power for these applications.

In recent years, ResNet [4], as the representative deep-network structure, has made breakthroughs in computer-vision tasks. Several well-known deep-learning networks, such as Faster R-CNN [5], Mask R-CNN [6], SSD [7], and YOLO [8,9,10,11,12,13,14], have provided effective solutions for object detection in most natural scenes. However, directly deploying these generalized networks to object detection is going to significantly degrade model performance in UAV remote-sensing images [15]. This is attributed to the unique characteristics of UAV remote-sensing images. Firstly, the significant variation in object scale due to the UAV’s altitude and viewing angle presents a major challenge. Objects close to the UAV appear large, while distant objects are significantly smaller, leading to extreme scale disparities. Furthermore, object density varies considerably, with frequent instances of object adjacency or occlusion. Secondly, the diverse periods captured by the UAV result in severe illumination variations, further compromising image quality. Consequently, directly applying mainstream object detectors to UAV remote-sensing data poses significant hurdles.

**Figure 1:**
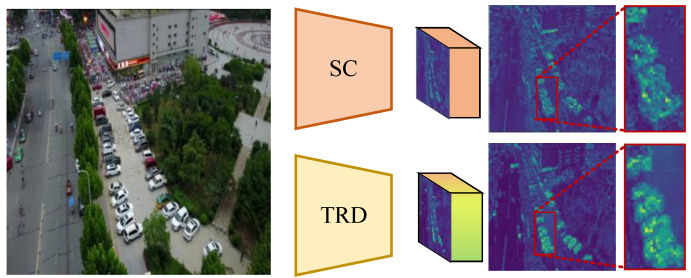
Visualization of the role of strided convolution (SC) with TRD in an RGB image. The output visualization is the result of maximizing the downsampling feature map along the channel direction. TRD’s activation pattern is more subtle and extensive. It shows that it preserves object gradients better than traditional downsampling during resolution reduction.

Currently, mainstream object detectors usually utilize a progressively smaller feature pyramid as a backbone network to extract a large number of visual features, thus reducing the computational load of the network to process large-scale feature maps. Backbone streamlines feature representations through downsampling-layer (DSL) operations to expand the neural sensory field of deeper networks, thereby enabling the construction of deeper networks, such as ResNet [4], VGG [16], and RegNet [17]. DSL typically adopts a convolution operation with a stride of 2. This operation has been proven to be effective in streamlining local feature information. However, it also inevitably leads to the loss of valuable information. As shown in Figure 1, compared to the method in this paper, simple strided convolution (SC) is more likely to produce blurring of features such as object boundaries and textures. It leads to difficulties in obtaining effective object discriminative features.

**Figure 2:**
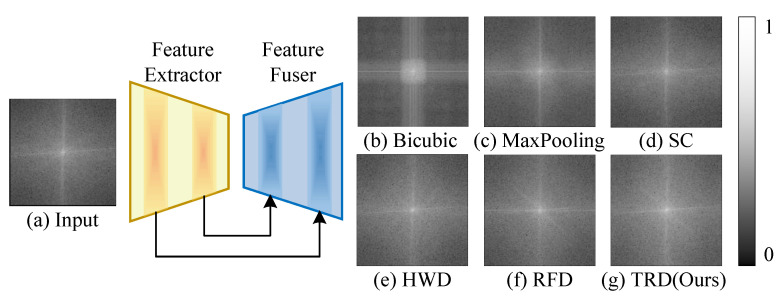
Comparison of the spatial frequency spectrum of an image after different downsampling and generalized upsampling feature fusion. The center represents low frequencies, the corners represent high frequencies, and the white areas indicate signal strength at each frequency. It indicates that simple downsampling leads to the loss of many detailed high-frequency features, which causes irreversible damage to subsequent feature fusion.

The above problem is particularly obvious in object detection of UAV images. Images captured by UAVs contain a large number of multi-scale, dense targets, which necessitate richer and more detailed semantic information for accurate image understanding. However, conventional downsampling methods lead to information loss, and feature blurring [18], especially explicit shallow features (boundaries, textures, etc.). As shown in (b) and (c) of Figure 2, simple downsampling struggles to generate feature responses to high-frequency regions, implying that a significant number of detailed high-frequency features are lost. DSL employing SC only in (c) of Figure 2 may result in the loss of crucial semantic information, thus reducing the detector’s performance. Although the subsequent feature fusion layer improves feature utilization to some extent, recovering the largely lost features through subsequent layers is difficult to achieve. It becomes a significant bottleneck limiting object-detection accuracy.

To mitigate the above problems, we propose a novel lightweight downsampling module named TRD. The core idea is to model feature reconstruction for SC downsampling as a task of feature super-resolution recovery. First, we iterate upsampling and downsampling on a given feature map to compute the residuals during SC based on back-projection. The back-projection, which is an efficient iterative process for minimizing reconstruction errors, has been used with remarkable results in super-resolution tasks [19]. Next, the residuals are used to guide the reconstruction of the downsampled ideal feature map. This operation aims to establish a good nonlinear relationship between the reconstructed features and subsequent feature learning. Finally, TRD uses dynamic weights within its back-projection unit to iteratively minimize feature loss during the downsampling process. In this way, more rich and effective shallow semantic information is dynamically provided to the deep network during the iterative learning process. Consequently, TRD demonstrates robust generalization across varying model sizes, data scenarios, and other influencing factors, leading to an improvement in the overall performance of the object detector.

In summary, TRD is essentially a dynamic feature restoration for the SC downsampling feature maps, which reconstructs the defects of SC downsampling. This work contributes to the development of more efficient object-detection methods in UAV remote-sensing images. TRD has the potential to overcome the accuracy limitations currently hindering the performance of mainstream object detectors when applied to aerial datasets. Specifically, our experimental results show that various types of network models equipped with TRD obtain an average 1∼3% AP boost compared to the baseline, with good model generalization. Moreover, TRD does not add a too heavy computational resource overhead. The work in this paper is summarized as follows:

- We propose an inverse projection-based sample residual learning structure that computes the projection error to guide the reconstruction of the downsampled ideal feature maps by cascading the upsampling and downsampling operators.
- The proposed TRD method mitigates the inherent trade-off between resolution reduction and information loss. It effectively improves the multi-scale object-detection accuracy in UAV remote-sensing images.
- Extensive experiments are conducted with the TRD on VisDrone-DET and NWPU VHR-10 datasets, showing that TRD can improve the multi-scale detection performance of the model compared to the baseline with little additional cost.

## 2. Related Work

### 2.1. Object Detection in UAV Remote-Sensing Images

The deep neural network-based detector achieves advanced performance in generalized natural scenes. Due to the randomness of the UAV’s camera angle and altitude, UAV remote-sensing images exhibit a larger field of view, denser small objects, and more complex illumination compared to natural scene images [20,21,22]. The direct application of general-purpose object detectors to object detection in UAV remote-sensing images can lead to a drastic degradation of detector performance [23].

To solve these problems, Xian et al. [24] proposed a feature fusion method based on NSNP-type neurons to help solve the problem of lost edge detail information. Similarly, Ren et al. [25] proposed a new dual-attention guided multi-scale feature aggregation network that can obtain more discriminative feature representations. Concurrently, researchers have explored utilizing contextual information [26] and super-resolution [27] to enhance feature discriminativeness for subsequent network layers. Bhowmik et al. [28] proposed a context-likelihood graph based on object distribution prior and refined it using network iterations. This results in a more accurate representation of the image. Zhang et al. [29] used assisted super-resolution to learn high-resolution feature representations to distinguish small objects from large backgrounds. It is usually used to fine-tune the recovery of small-scale objects, which helps the detector to obtain fuller discriminative features.

### 2.2. Downsampling in CNNs

Downsampling is a crucial component of deep convolutional neural networks, serving to adjust the resolution of input feature maps, broaden the receptive field, and decrease the computational load. The main downsampling operations commonly used are maxpooling [30], average pooling, and SC [31].

The pooling operation is based on the mathematical relationship of the domain for targeted selection and does not have any learned parameters [32]. Therefore, simple pooling operations lead to the loss of important spatial information and cannot adapt to the complexity of multi-scenarios. To address these challenges, AdaPool [18] introduces an adaptive exponential weighting scheme for information feature preservation pooling. It has better network connectivity and can adaptively capture details. Wavelet pooling [33] introduces wavelet variations by two-level feature decomposition and discarding one level to reduce the feature dimension. This operation effectively mitigates the overfitting problem in the image classification problem. SoftPool [34] preserves the structure of pooling while also minimizing the loss of information during the maxpooling process, further improving detection accuracy.

Simple SC can cope with object detection in most natural scene images but still has limitations in facing small and dense objects as well as more complex and specific scenes. To address this, RFD [35] extracts a more robust downsampling feature map by fusing features from multiple downsampling methods, enabling subsequent feature learning to better capture key information and improve the overall performance of the network. Similarly, Hesse et al. [36] proposed an adaptive downsampling scheme that allows different feature map regions to be processed at different resolutions for better computer-vision tasks. HWD [32] also applies wavelet transform to reduce the spatial resolution of the feature maps and, at the same time, retain as much information as possible. This operation effectively improves the segmentation performance of CNN in computer-vision tasks. The above research results show that the ability of downsampling to retain information has a critical impact on model performance. In other words, the retained information needs to dynamically respond to subsequent layers to learn better representational features.

### 2.3. Back-Projection in CNNs

The back-projection (BP) algorithm is an effective approach to minimize the reconstruction error [37] and is widely used in image super-resolution (SR) tasks and low-light enhancement tasks with remarkable results. BP is designed to provide differential feedback to the optimization process by iteratively back-projecting upsamplings and downsampling [38]. The focus of BP is to compute the error generated by the back-projections to guide the reconstruction of better results [39].

BP algorithm performs well in many computer-vision tasks. In the SR task, for a given low-resolution (LR) image, the quality of SR can be dynamically adjusted by iterating the BP block to effectively minimize the loss between LR and downsampled SR. Deep Back-Projection Network [40] constructs interconnected sampling stages, which allows the projection network units to functionally cascade and improves the reconstruction effect of SR. BP mechanism also shines in low-light enhancement tasks. Wang et al. [41] modeled low-light enhancement as a residual learning problem. Based on the BP mechanism, LBP blocks are proposed to iteratively learn the residuals of normal light estimation to achieve low-light enhancement. DCPT [42] produces darkness-resistant capability coded cues by introducing a back-projection structure, which achieves similar tracking performance in the dark as in the day with a small number of parameters. Park et al. [43] proposed a U-shaped BP enhancement network that balances the light information of the image. SRBPSwin [44] enhanced the feature-extraction capability of the network by combining BP and Swin transforms, which provided bidirectional feedback for the reconstruction error.

The above research results show that BP has mature theoretical support and experimental results in the field of texture information reconstruction. Downsampling in the field of object detection is a process of streamlining features accompanied by much information loss. Therefore, introducing a BP mechanism for the downsampling block can effectively mitigate these inherent limitations. This operation helps the network extract more detailed texture features, leading to better object-detection accuracy in UAV remote-sensing images.

## 3. Method

In this section, we present the principles and architecture of the proposed TRD in detail. First, we briefly describe the main points of the problem to be solved and the areas where TRD plays a role in object detectors in the overview of Section 3.1. Next, back-projection-based modeling of sampled residual learning is illustrated in Section 3.2. Finally, we illustrate the composition of TRD with specific implementation steps in detail in Section 3.3.

### 3.1. Overview of Additive TRD

Objects in UAV aerial images have unique scale variations, illumination variations, and variations in object densities, which pose unique challenges to the object-detection task. In particular, there are a large number of low-resolution, small-sized objects in these images, which often have only a small number of features. DSL is a key component of mainstream object detectors for aggregating features, expanding the receptive field, etc. However, simple downsampling tends to lose many key features, leading to spatial degradation of the object.

To mitigate this problem, we propose a novel dynamic texture-enhanced downsampling module named TRD based on back-projection, which is a plug-and-play module. We directly replace the SC downsampling of 2∼5 levels in the backbone network of detectors with TRD. TRD can dynamically supplement the effective gradient information of the object during the downsampling process, which provides rich feature information for the subsequent network from the root.

### 3.2. Sample Residual Learning Structure

DSL is a fundamental and extremely critical network layer in object detectors based on CNN. The purpose of DSL is to create compressed feature representations that aggregate valid feature information. However, it is difficult to reduce the resolution while keeping the effective information of the features intact. This is because there exists no uniquely defined valid sampled feature map $F_{out}$ for a given feature map $F_{in}$. For this reason, we define a novel sample residual learning structure for object-detection tasks based on back-projection.

**Figure 3:**
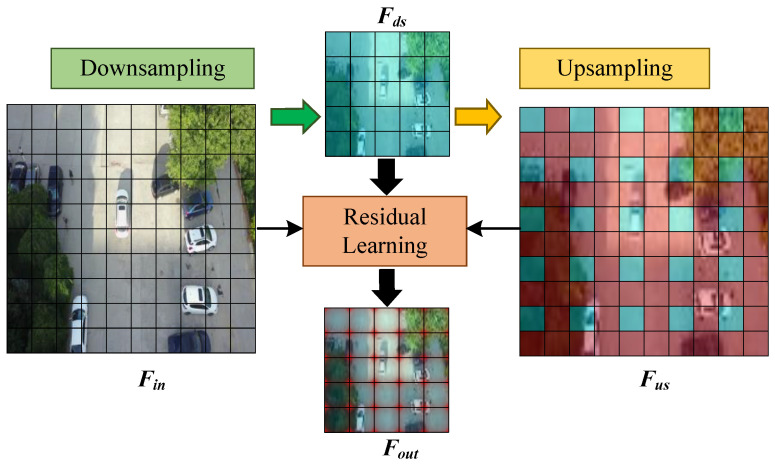
Implementation of the sample residual learning structure. In short, we can dynamically reconstruct texture features lost during downsampling by iteratively refining the projection error between downsampling and upsampling. This makes the feature map rich in discriminative features even after downsampling.

Sample Residual Learning Stages. The core of the structure is to guide the reconstruction of the ideal downsampled feature map by learning the back-projected residuals of the upsampled and downsampled blocks. The principle is shown in Figure 3. First, we can upsample the downsampled feature $F_{ds}$ based on back-projection to obtain the feature $F_{us}$ with the same scale as the input feature $F_{in}$. Upsampling aims to obtain the feature loss in the downsampling process. Then, we construct this loss as a kind of back-projected residual information *R*. Since *R* can expose the weakness of simple downsampling, we focus on learning the features of *R* during the sampling process. Furthermore, if downsampling produces the desired downsampling feature $F_{ds}$, the subsequent residual reconstruction will be weakened. Finally, the dynamically learned back-projection residual feature $F_{rl}$ is obtained by point-by-point weight assignment to the back-projection residual information *R*. Based on the residual information *R*, it can reconstruct the residual feature $F_{rl}$ by adding it to the base downsampling feature $F_{ds}$.

Analysis and Description. The back-projection process of upsampling and downsampling can be understood as a self-correcting process consisting of two main aspects. On the one hand, the mechanism combines the back-projection residuals with the surrounding contextual information and iteratively minimizes the residuals through feedback. On the other hand, the mechanism can obtain the nonlinear relationship of the sampling transform by learning the back-projection residuals of the upsampling and downsampling, and thus reconstructing more robust downsampling features $F_{out}$. The mechanism can be mathematically modeled as:

(1) $$F_{out}=L1\left(F_{ds}\right)+L2\left(F_{us}-L3\left(F_{in}\right)\right),$$

where $F_{us}$ denotes the feature map after upsampling the $F_{ds}$. L(·) denotes the dynamic weights for residual learning.

In summary, unlike directly learning the sampled mapping function, the proposed sample residual learning structure refines the high-frequency texture features of the object through an iterative projection process. In this process, the feature loss during the sampling process is continuously constructed through the back-projection unit, and various upsampling and downsampling operators are learned to preserve the high-frequency texture information. Thus, the sample residual learning structure can effectively address the substantial loss of object features caused by the backbone after multi-layer downsampling, providing sufficient effective features for subsequent feature fusion and network tasks.

### 3.3. Texture Reconstructive Downsampling

**Figure 4:**
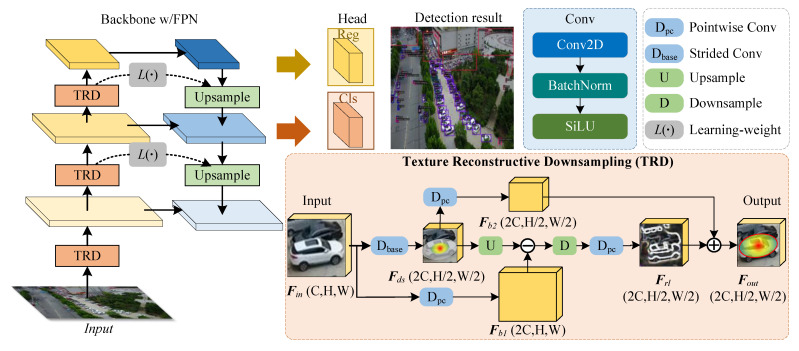
Structure of Texture Reconstructive Downsampling (TRD). The C, H, and W denote the channel dimension, height, and width of the feature map, respectively. TRD aims to address the irreversible information loss that occurs during conventional downsampling. TRD consists of three core designs: (1) Using efficient SC as the basis for downsampling; (2) Using pointwise convolution as a balancing weight to mitigate semantic conflicts in feature fusion. (3) Dynamically reconstructing for texture features lost by iteratively learning the back-projection residuals during upsampling and downsampling.

The structure of the texture reconstructive downsampling module (TRD) is shown in Figure 4. TRD consists of three core designs: (1) Using SC as the base feature; (2) Using pointwise convolution as the balancing weight to reduce semantic conflicts in feature fusion; (3) Using sample residual learning structure to reconstruct lost texture features. We have specified these steps below.

Generating Reference Sampling Features. Experiments have demonstrated SC’s effectiveness in aggregating features and expanding the receptive field. Many previous studies, such as ResNet and YOLO, use efficient downsampling approaches with SC. Here, we apply SC to a given feature map *x* to obtain a base downsampling feature map that has filtered a lot of redundant information and retained more critical information. We name this process $D_{base}$, which is responsible for increasing the channel to the subsequent number of input channels and transforming the scale to 1/2 of the input scale. $D_{base}$ can be expressed by a mathematical relation as:

(2) $$\begin{array}{cc} \mathrm{Conv} & =\mathrm{SiLU}(\mathrm{BN}(\mathrm{Conv}2D(x)), \end{array}$$

(3) $$\begin{array}{cc} D_{base} & ={\mathrm{Conv}}_{\left(3,2\right)}\left(x\right), \end{array}$$

where SiLU(·), BN(·), Conv2D(·) denote SiLU activation function, batch normalization and 2D convolution, respectively. ${\mathrm{Conv}}_{\left(3,2\right)}\left(\cdot \right)$ denotes the convolution module operation with a convolution kernel size of 3 × 3 and stride of 2. The input feature $F_{in}$ undergoes $D_{base}$ operation to obtain the base downsampling feature map $F_{ds}$.

Mitigating Cross-layer Semantic Conflicts. Semantic conflicts are mitigated using pointwise convolution. DSL performs a 2X reduction of the original scale. The TRD process involves multiple feature fusion steps, requiring careful consideration of potential noise and conflicting features. Furthermore, the dynamic nature of the back-projection structure arises from its iterative refinement of features through the analysis of residuals from the projection process. Therefore, we incorporate pointwise convolution into the fusion process of several key features to perform similar “encoding” and “decoding” operations on the feature maps. We name this method $D_{pc}$, which can be expressed as a mathematical relationship:

(4) $$D_{pc}={\mathrm{Conv}}_{\left(1,1\right)}\left(x\right),$$

where ${\mathrm{Conv}}_{\left(1,1\right)}\left(\cdot \right)$ denotes a convolution module with a convolution kernel size of 1 × 1 and a stride of 1, and *x* denotes a given feature. The operation chooses pointwise convolution for two reasons. On the one hand, the number of parameters brought by pointwise convolution is extremely small, which is conducive to the lightweight of the network. On the other hand, pointwise convolution can model the inter-neighborhood feature relationship of pixels and learn the residual contribution of different regions, thus balancing the residual learning in the iterative process of TRD.

Reconstructing Texture Features. The sample residual learning structure is the core part of TRD, which dynamically refines the high-frequency texture features of an object through an iterative projection process. First, we clone the input feature map into two maps. One is processed by SC to obtain the base downsampling feature $F_{ds}$, which reduces the spatial dimension by a factor of 2. One is processed by pointwise convolution to obtain $F_{b1}$, which extends the number of channels of the feature map. Pointwise convolution can avoid feature conflicts during feature interaction and balance residual learning. Next, we obtain $F_{us}$ by inversely upsampling the feature map $F_{ds}$ that has been downsampled by SC. It yields the residual information *R* between the upsampled feature map $F_{us}$ and the input feature map $F_{in}$. This residual term *R* somewhat reflects the weakness of conventional downsampling. Then the average pooling operation is performed on the residual information between $F_{b1}$ and $F_{us}$. The experimental results show that average pooling facilitates the residual information to be added to the underlying downsampling features without introducing too many parametric quantities and computations. This is because average pooling in additive residuals can retain richer gradient information. At last, the downsampling feature map $F_{ds}$ and the residual information *R* are learned iteratively by pointwise convolution on the residual information, respectively. The process we named $D_{res}$, which can be expressed mathematically as:

(5) $$\begin{array}{cc} F_{ds} & =D_{base}\left(F_{in}\right), \end{array}$$

(6) $$\begin{array}{cc} D_{res} & =D_{pc}\left(D\left(U\left(F_{ds}\right)-D_{pc}\left(F_{in}\right)\right)\right), \end{array}$$

where $F_{in}$ denotes the input features, U(·) denotes an upsampling operation, and D(·) denotes an average pooling operation.

Forming the TRD Module. The residual-learned feature maps and the base downsampling feature maps are summed point by point to obtain the augmented downsampling feature maps $F_{out}$. If the downsampling is ideal enough, the learned residual features $F_{rl}$ will not have any effect on the base $F_{ds}$ because the $F_{ds}$ already have enough information at this point. The TRD can be expressed mathematically as:

(7) $$\begin{array}{cc} TRD & =D_{res}\left(F_{ds}\right)+D_{pc}\left(F_{ds}\right), \end{array}$$

(8) $$\begin{array}{cc} F_{out} & =TRD(F_{in}), \end{array}$$

where $F_{in}$ denotes the input features and $F_{out}$ denotes the output features by TRD.

Furthermore, we compare the visualization differences between SC and the proposed TRD in Figure 1. It can be seen that the proposed TRD has richer texture information compared to SC downsampling. Analyzing human visual perception, it is obvious that the output of TRD has richer texture information of the object. In addition, Figure 2 shows the spectrum of TRD versus other downsampling methods. It can be seen that the high-frequency response of conventional downsampling is seriously inadequate, in contrast to TRD, which has a superior full-frequency response. In conclusion, the proposed TRD module dynamically introduces effective feature information. Thus, it refines the detailed high-frequency texture features of the object and effectively mitigates the feature loss due to resolution reduction.

## 4. Experiments

### 4.1. Experiment Setting

**VisDrone-DET dataset.** This dataset is acquired from various UAV cameras in different scenes, different weather and lighting. The dataset is mainly used for object detection and consists of 288 video clips and 10,209 high-resolution UAV images. The training set consists of 6471 images, the test set consists of 3190 images, and 548 images from the validation set. Image resolutions range from 960 × 540 to 2000 × 1500, and the dataset showcases complex scenes covering 10 different object classes and localization frames. The dataset contains objects with significant scale variation and category imbalance, making it an ideal experimental specimen for studying the challenges of small object detection. The dataset metrics validated in this paper are based on the validation dataset in this dataset.

**NWPU VHR-10 Dataset.** This dataset is a public challenging remote-sensing object-detection dataset. The image resolutions cover the range from 500 × 500 to 1100 × 1100, containing a large number of complex scenes covering 10 different kinds of object classes. The image resolutions cover the range from 500 × 500 to 1100 × 1100, containing a large number of complex scenes covering 10 different kinds of object classes. The average size of these ten classes of objects accounts for about 6.4% of the image size.

**Implementation Details.** The specific experimental setup of this paper is described below. We implemented our method based on PyTorch 1.12.1. All experiments were performed on a server with the system Ubuntu 18.04.6 LTS. The server contains 8 NVIDIA GeForce RTX 3090 (24 GB). To ensure the fairness and consistency of the experiments, all experiments and benchmark model replications in this paper were conducted in MMYOLO. Among them, the batch size was set to 16, the number of training rounds was set to 300, and the input image size was fixed to 640 × 640 for all networks of the YOLO series during the training phase. Unless otherwise specified, all network parameters adopted the default settings in MMYOLO. In addition, the evaluation metrics we used follow the format defined by the MS COCO dataset. AP, ${\mathrm{AP}}_{50}$, ${\mathrm{AP}}_{75}$, ${\mathrm{AP}}_{S}$, ${\mathrm{AP}}_{M}$, and ${\mathrm{AP}}_{L}$. The ${\mathrm{AP}}_{S}$, ${\mathrm{AP}}_{M}$, and ${\mathrm{AP}}_{L}$ denote small-scale, medium-scale, and large-scale AP results, respectively. The defined object scales are not. The small size has an area less than 32 × 32, the medium size has an area between 32 × 32 and 96 × 96, and the large size has an area greater than 96 × 96.

### 4.2. Overall Performance of TRD

Detection Results on VisDrone-DET dataset. To further demonstrate the state-of-the-art performance of the proposed method, we applied TRD and mainstream object detectors to the VisDrone-DET dataset. The detectors mainly include two-stage models, one-stage models, and recent advanced models. The selection criteria were code availability and test datasets with the same evaluation metrics. Then, we assessed the performance difference between the proposed method and current advanced models. The experimental results, shown in Table 1, indicate that the detectors with TRD achieved performance improvements. Specifically, YOLOv5-X improved by 1.9% AP, and YOLOv8-X improved by 3.0% AP. YOLOv8-X with TRD achieved state-of-the-art detection accuracy of 31.0% AP. Notably, TRD generally improves the detection accuracy of small-scale, medium-scale, and large-scale objects. This indicates that TRD can mitigate information loss for objects of different scales during downsampling, effectively improving the multi-scale object-detection capability of the detectors.

Detection Results on NWPU VHR-10 dataset. Similarly, multiple models equipped with TRD achieved substantial gains on the NWPU VHR-10 dataset. Compared with the strong baseline RTMDet, the RTMDet equipped with TRD obtains an improvement of 2.2% AP and comprehensively improves the AP of each category. The comparative results in Table 2 demonstrate TRD’s advanced generalization capability, enhancing the object-detection accuracy of multiple models.

**Figure 5:**
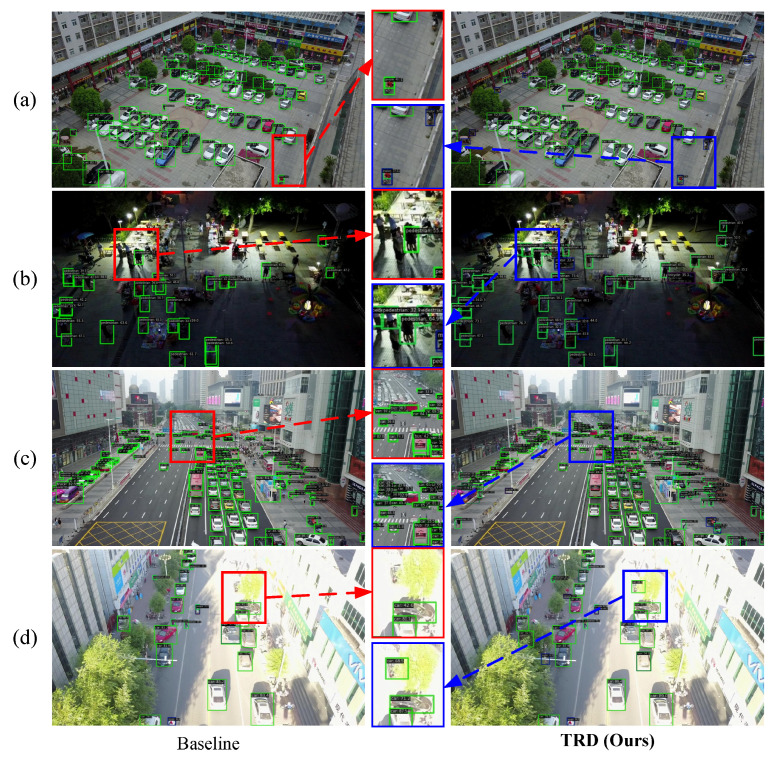
Visualization of Detection Results on the VisDrone-DET Dataset. (**a**–**d**) denote the images with different lighting conditions, scale distributions, and densities, respectively. The red box indicates the detection effect of YOLOv8-X, and the blue box indicates the detection effect of YOLOv8 equipped with TRD. It can be seen that TRD helps the model to drastically reduce instances of misdetections and missed detections in a variety of harsh conditions. It suggests that TRD is effective in improving model generalization.

**Figure 6:**
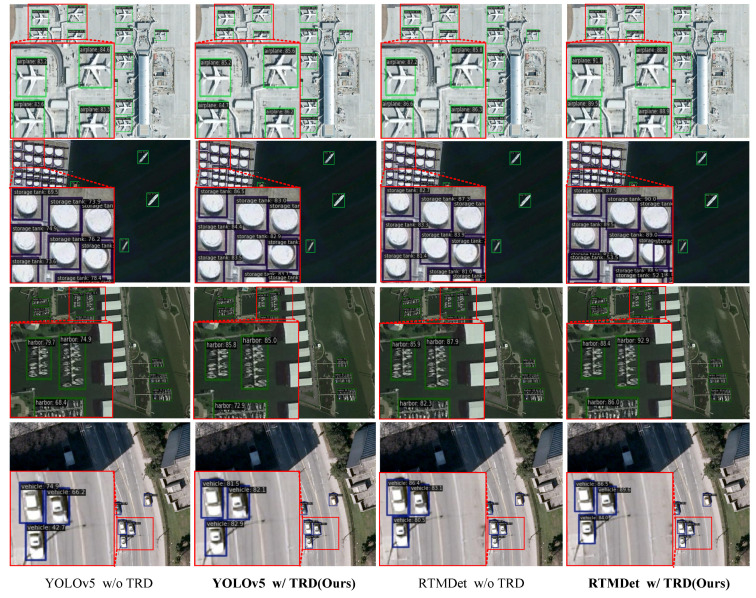
Visualization of Detection Results on the NWPU VHR-10 Dataset. The role of TRD is quantitatively analyzed using YOLOv5 and RTMDet as baselines, respectively. It can be seen that TRD improves the confidence of the model on the true object. This indicates that TRD can effectively improve the accuracy and recall of the model.

Visualization of Detection Results. Several visualizations of the VisDrone-DET and NWPU VHR-10 datasets are shown in Figure 5 and Figure 6, respectively. The comparative detection results in Figure 5 show that introducing TRD reduces the number of missed and false detections compared to the baseline. In particular, TRD exhibits better detection performance for objects of different scales in various environments. Meanwhile, Figure 6 shows that TRD improves the model’s confidence in detecting various targets, indicating that TRD enhances the model’s target robustness. The above experiments demonstrate that the feature enhancement TRD provides to the model is a generalized feature, effectively improving the model’s detection performance.

**Figure 7:**
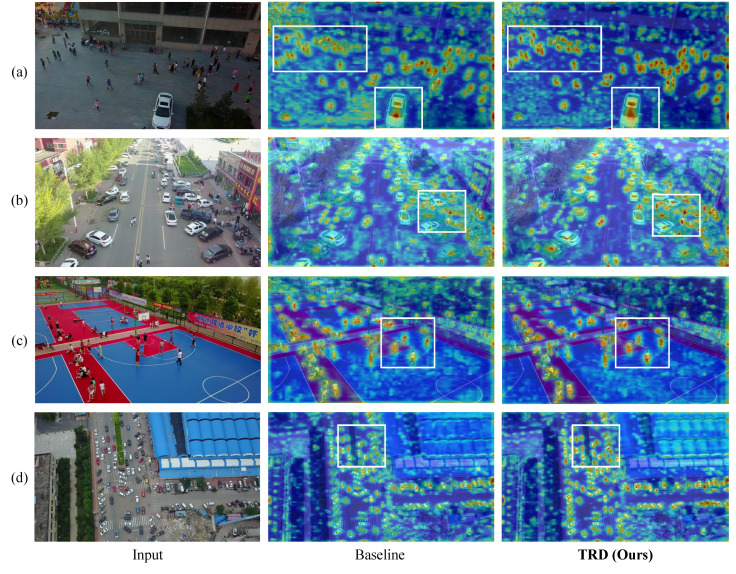
Visualization of AblationCAM Results. (**a**–**d**) denote the images from four different UAV views, respectively. We visualize AM information by normalizing the contribution of each channel. It can be seen that TRD can provide a more comprehensive activation region for object detection. It suggests that TRD can extract richer discriminative features to help the detector recognize objects more subtly.

AblationCAM Visualization Results with TRD. AblationCAM uses ablation analysis to visualize the importance of individual feature mapping units. It can localize to the detection box region to help explain the model’s prediction process. As observed in Figure 7, the incorporation of TRD allows the model to delineate clearer and more confident boundaries for each class (red regions), and it also suppresses partial spurious activations in background regions. It strongly suggests that TRD introduces more discriminative texture features during model training, providing more reliable contributing regions.

**Figure 8:**
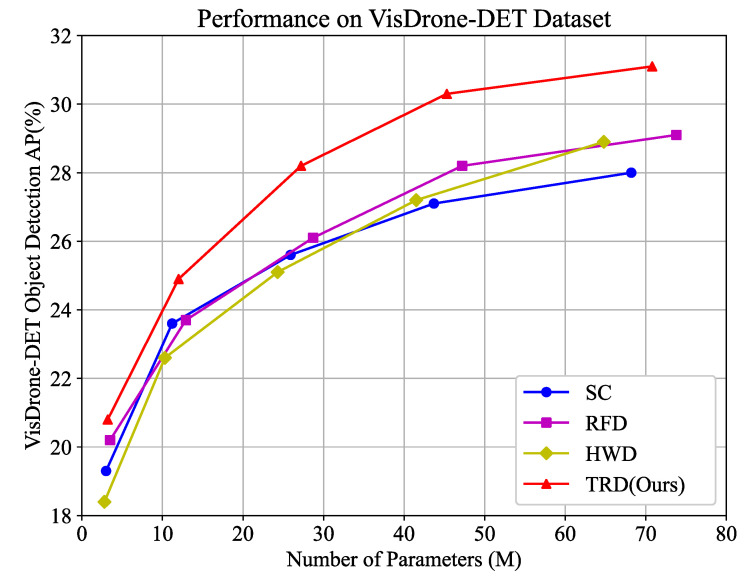
Comparison in terms of size-accuracy trade-off on VisDrone-DET dataset. TRD shows great advantages over other advanced downsampling methods. (1) Higher accuracy. (2) Higher parameter utilization. (3) Better model size adaptation.

Comparison of Different Model Sizes with TRD. Mainstream object detectors employ different training strategies for models of varying sizes. A single component may exhibit differing performance across these model sizes. To verify TRD’s adaptability to different network model sizes, we replaced the SC downsampling in the N/S/M/L/X networks of YOLOv8 with TRD. The experimental results, shown in Table 3, clearly indicate that TRD can improve the baseline network’s AP by 1∼3%. Specifically, TRD yields a substantial 3.0% AP improvement for YOLOv8-X, demonstrating the effectiveness of the proposed method. To further demonstrate the superiority of the proposed method, we compared TRD against other state-of-the-art downsampling methods. The test results are shown in Figure 8. It can be seen that the balanced performance with TRD in terms of parameters and performance is ahead of the other methods, which indicates that TRD substantially improves the efficiency of the network parameters. It can be noticed that the contribution of TRD to the baseline network is becoming stronger in the face of increasing model size. It indicates that TRD can help the deep network to mine richer discriminative features. Moreover, TRD delivers significant enhancement in the face of different model sizes and objects of different scales. It indicates that the texture features enhanced by TRD are general features belonging to object detection with strong generalization.

At the same time, we also report the additional computational cost due to TRD in Table 3. It can be observed that TRD enables the baseline to attain comparable accuracy to a larger-size model. For example, YOLOv8-M equipped with TRD achieves an accuracy of 28.2% AP, which is comparable to the 27.1% AP of YOLOv8-L. It suggests that the TRD can provide an improvement in parametric efficiency. Similarly, YOLOv8-X equipped with TRD brings an improvement of 3.0% AP. It only increases the computational cost by about 5.0%, specifically by 2.5 MB of parameters and 7.3 GFLOPs of computation. TRD performs better size-precision trade-offs. Consequently, we posit that TRD effectively mitigates the deficiency in high-frequency texture representation characteristic of traditional downsampling techniques. This superior preservation of fine-grained texture features leads to a marked improvement in object-detection accuracy.

**Figure 9:**
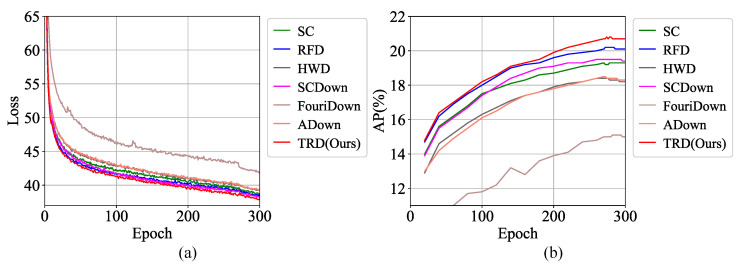
Comparison of loss and AP values on the VisDrone-DET dataset. (**a**) Represents the comparison results of loss values; (**b**) Represents the comparison results of AP values. We use YOLOv8-N as a baseline. Compared to other state-of-the-art downsampling methods, TRD demonstrates superior performance. It exhibits the lowest loss values and a faster convergence rate during training while consistently maintaining the highest detection accuracy. These results indicate that TRD has superior feature-extraction performance.

Comparison of Different Downsampling Methods. We evaluate the performance of various mainstream downsampling methods on object detectors. Specifically, these downsampling methods and TRD are deployed on the DSL of YOLOv8-N for comparative testing. SC is the initial DSL of YOLOv8. We present the results on the VisDrone-DET validation dataset in Table 4. Compared to other downsampling methods, TRD’s object-detection performance is state-of-the-art, achieving a 1.5% AP and 2.2% ${\mathrm{AP}}_{50}$ improvement over the original SC. Using YOLOv8-N as the baseline, iteration information for different downsampling methods is shown in Figure 9. We can find that TRD can speed up the convergence of the loss and further reduce the loss. At the same time, the growth and the final value of the AP of TRD are much larger than the other methods. Collectively, these data suggest that TRD effectively extracts richer discriminative features, accelerates network convergence, and improves model accuracy.

### 4.3. Ablation Study

Analysis of Components of TRD. We performed ablation experiments on the internal components of the TRD module, using YOLOv8-N as the baseline. The interior of TRD can be divided into three main processes: $D_{base}$, $D_{res}$, and $D_{pc}$. In particular, TRD degrades to SC when $D_{res}$ and $D_{pc}$ are not applied, and internal use of $D_{pc}$ without $D_{res}$ is equivalent to the direct assignment. We report the overall ablation results in Table 5, revealing that the $D_{res}$ structure contributes 66.6% of the AP, unlike the attention mechanism that may give extra strategies to small objects and reduce the accuracy of other scale objects. $D_{res}$ improves the accuracy of small, medium, and large object detection across the board. It indicates that $D_{res}$ has better scale invariance. Meanwhile, $D_{pc}$ can mitigate semantic conflicts at different levels, further improving the detection performance of TRD. Notice that the computational resource consumption of components of TRD is low, indicating that the proposed method is lightweight.

Analysis of Different Downsampling inside TRD. It is observed that the core functionality of TRD is closely related to the downsampling method during the $D_{res}$ process. This step demonstrates the ability of TRD to learn high-frequency texture features for object details. Therefore, we further explore the effect of different downsampling methods on the performance of TRD using the YOLOv8-S model as a baseline. The results are shown in Table 6. As can be seen, TRD is more friendly to large-core convolution, leading to a 2.2% AP improvement. However, large kernel convolution introduces a larger computational cost. On the contrary, average pooling yields a 1.3% AP gain with a negligible computational overhead of only 0.8 MB. The $D_{res}$ operates on residual information, and average pooling can provide smooth gradients for minimizing reconstruction errors. Therefore, we choose average pooling as the downsampling in $D_{res}$ to provide a more balanced performance.

## 5. Conclusions

In this work, we propose TRD, a lightweight and efficient downsampling module designed for multi-scale object detection in UAV remote-sensing images. Convolutional downsampling can aggregate local information but tends to result in dropping the internal texture of objects. It is extremely unfavorable for remote-sensing images, which are characterized by objects with large-scale variations and uneven densities. Therefore, the proposed TRD module effectively alleviates the above problems. Unlike the conventional attention mechanism module and redundant convolutional stacking, TRD is a simple and efficient feedback-based reconstruction process. TRD reconstructs more efficient texture features by minimizing the projection error of the sampling process, which reduces the loss of critical information caused by conventional downsampling. We evaluated the TRD module on multiple networks with extensive experimental and ablation studies on the VisDrone-DET and NWPU VHR-10 datasets. The results show the effectiveness and generalizability of the TRD module. Specifically, the TRD module has two major advantages. On the one hand, as a plug-and-play module, TRD can directly and seamlessly replace existing downsampling modules with little additional cost. On the other hand, TRD significantly improves object-detection performance.

Since downsampling is a general module, the proposed TRD module still has significant generalization potential for computer-vision tasks such as classification, segmentation, and image enhancement. The main limitations of the TRD module are its efficiency and localization. First, a large kernel convolution significantly boosts TRD performance, but it comes with the cost of increased parameters. Our goal is to improve the efficiency of TRD by introducing a larger receptive field using a less computationally expensive convolution strategy. Second, due to the lack of global information captured by convolution operations, it is difficult for TRD to capture global spatial relationships, resulting in overly localized effects. We will consider incorporating global information and ensuring proper spatial alignment for TRD. In the future, we aim to further refine TRD, contributing to generalized computer-vision tasks. We hope this work will inspire more research to enhance the feature-extraction capabilities of backbone networks.

## Figures and Tables

**Table 1:** Comparison results for the VisDrone-DET dataset under the COCO standard. “-” indicates that the original paper did not provide data. Bold print highlights the best results.

Method	Venue	AP (%)	AP_50_ (%)	AP_75_ (%)	AP_S_ (%)	AP_M_ (%)	AP_L_ (%)
FreeAnchor [45]	TPAMI2022	20.0	33.6	20.9	11.4	30.0	36.7
SDPNet [46]	TGRS2023	30.2	**52.5**	30.6	-	-	-
CCOD [47]	TIM2022	20.9	35.8	21.1	12.3	31.3	35.1
FPSOD [48]	TGRS2024	23.1	38.0	24.1	15.1	34.2	36.8
DTSSNet [49]	TGRS2024	25.5	41.1	26.9	18.6	34.3	41.2
BRSTD [50]	TGRS2024	27.3	45.9	-	-	-	-
QueryDet [51]	CVPR2022	28.3	48.1	28.8	-	-	-
ToMe [52]	CVPR2023	27.8	46.9	28.5	-	-	-
RFLA [53]	ECCV2022	27.4	45.3	-	-	-	-
HIC-YOLOv5 [54]	ICRA2024	25.9	44.3	-	-	-	-
YOLOv5-X	Ultralytics2022	22.6	38.6	21.8	13.9	32.4	42.6
CEASC [55]	CVPR2023	20.8	35.0	31.5	-	-	-
YOLOv7 [11]	CVPR2023	27.8	49.2	27.5	18.6	38.8	47.8
YOLOv8-X	Ultralytics2023	28.0	45.4	26.8	16.7	38.9	45.5
YOLOv5-X w/TRD (Ours)	-	24.5	40.9	24.8	15.2	36.2	42.7
**YOLOv8-X w/TRD (Ours)**	-	**31.0**	49.5	**32.3**	**19.8**	**45.7**	**59.4**

**Table 2:** Comparison results for the NWPU VHR-10 dataset under the COCO standard. “*” denotes the results of our implementation. The networks tested are the minimum models. Bold print highlights the best results. The abbreviations stand for airplane (AI), ship (SH), storage tank (ST), baseball diamond (BD), tennis court (TC), basketball court (BC), ground track field (GTF), harbor (HA), bridge (BR) and vehicle (VE). The AP(%) was taken as the metric for each category.

Method	Backbone	Category (%)										AP (%)
		AI	SH	ST	BD	TC	BC	GTF	HA	BR	VE	
RetinaNet [56]	ResNet-50	58.4	46.8	53.1	55.2	49.4	43.2	23.5	32.5	17.1	45.5	42.5
Faster R-CNN [5]	ResNet-50	63.9	54.7	**60.3**	58.5	61.3	64.1	35.6	41.3	33.5	59.6	53.3
RTMDet *	CSPNeXt	65.2	55.4	55.1	72.0	68.1	63.2	80.2	60.0	43.7	55.7	61.8
YOLOv5 *	Darknet	61.9	47.9	41.2	69.9	60.4	51.4	78.3	48.5	44.3	47.8	55.2
YOLOX [12]	Darknet	62.1	52.0	45.9	60.5	55.9	57.2	24.7	47.4	30.4	51.6	48.8
YOLOv7 [11]	Darknet	59.4	53.3	44.3	61.9	55.5	55.6	32.6	51.0	39.6	49.6	50.3
YOLOv8 *	Darknet	65.9	52.5	49.0	71.5	66.5	53.3	81.2	57.1	45.3	48.6	59.1
CR-Mixing [57]	Darknet	**75.9**	15.7	50.4	**85.2**	**91.1**	65.7	76.5	26.0	12.9	43.8	54.3
YOLOv5 w/TRD	Darknet	60.6	51.4	41.7	71.2	63.3	50.9	82.4	57.9	42.3	47.5	56.9
YOLOv8 w/TRD	Darknet	63.8	**55.9**	49.6	73.8	68.8	62.0	**83.6**	**60.3**	**46.6**	57.1	62.2
RTMDet w/TRD	CSPNeXt	66.7	55.7	55.6	74.1	72.1	**70.0**	83.4	58.4	43.8	**60.2**	**64.0**

**Table 3:** Results of the accuracy comparison between the YOLOv8 and the DSL of it replaced by the TRD module for different model sizes on the VisDrone-DET dataset. The Red indicates an increase in positive impacts and Blue indicates an increase in negative impacts.

Model	DSL	AP (%)	AP_50_ (%)	AP_75_ (%)	AP_S_ (%)	AP_M_ (%)	AP_L_ (%)	#Param (M)	GFLOPs
YOLOv8-N	SC	19.3	33.0	19.3	10.4	29.3	42.1	3.0	4.1
	**TRD**	20.8 (+1.5)	35.2 (+2.2)	21.0 (+1.7)	11.7 (+1.3)	31.8 (+2.5)	44.8 (+2.7)	3.2 (+0.2)	4.6 (+0.5)
YOLOv8-S	SC	23.6	40.0	21.8	12.7	33.4	42.0	11.2	14.4
	**TRD**	24.9 (+1.3)	41.0 (+1.0)	25.5 (+3.7)	14.8 (+2.1)	37.5 (+4.1)	45.6 (+3.6)	12.0 (+0.8)	16.3 (+1.9)
YOLOv8-M	SC	25.6	42.6	24.0	14.8	35.5	41.8	25.9	39.6
	**TRD**	28.2 (+2.6)	45.8 (+3.2)	29.3 (+5.3)	17.5 (+2.7)	42.3 (+6.8)	55.9 (+14.1)	27.2 (+1.3)	43.6 (+4.0)
YOLOv8-L	SC	27.1	44.1	24.8	15.3	36.0	44.7	43.7	82.7
	**TRD**	30.3 (+3.2)	48.6 (+4.5)	31.5 (+6.7)	19.8 (+4.5)	44.8 (+8.8)	56.0 (+11.3)	45.3 (+1.6)	89.4 (6.6)
YOLOv8-X	SC	28.0	45.4	26.8	16.7	38.9	45.5	68.2	132.1
	**TRD**	**31.0 (+3.0)**	**49.5 (+4.1)**	**32.3 (+5.5)**	**19.8 (+3.1)**	**45.7 (+6.8)**	**59.4 (+13.9)**	70.8 (+2.5)	139.4 (+7.3)

**Table 4:** Comparative results of different downsampling methods on the VisDrone-DET dataset. “*” denotes the results of our reimplementation.

Method	Venue	AP (%)	AP_50_ (%)	AP_75_ (%)	#Param (M)	GFLOPs
SC *	-	19.3	33.0	19.3	3.0	4.1
RFD [35] *	TGRS2023	20.2	34.6	20.0	3.5	6.3
HWD [32] *	PR2023	18.4	32.1	18.1	2.8	3.8
SCDown [14] *	ARXIV2024	19.5	33.4	19.2	2.7	3.8
FouriDown [58] *	NeurIPS2024	15.1	26.6	14.6	2.7	3.7
ADown [13] *	ECCV2024	18.5	31.8	18.2	2.7	3.7
**TRD**	-	**20.8**	**35.2**	**21.0**	3.2	4.6

**Table 5:** Ablation experiments with TRD on the VisDrone-DET dataset. “✔” indicates that the method is used, while “**×**” means the method is not used. It can be seen that the $D_{res}$ structure can bring half of the performance contribution. $D_{pc}$ can effectively mitigate semantic conflicts at different levels, further improving the detection performance of TRD.

$D_{base}$	$D_{res}$	$D_{pc}$	AP (%)	AP_50_ (%)	AP_75_ (%)	AP_S_ (%)	AP_M_ (%)	AP_L_ (%)	#Param (M)	GFLOPs
✔	**×**	**×**	19.3	33.0	19.3	10.4	29.5	42.3	3.0	4.1
✔	✔	**×**	20.3	34.6	20.4	11.1	31.1	44.2	3.1	4.3
✔	✔	✔	**20.8**	**35.2**	**21.0**	**11.7**	**31.8**	**44.8**	3.2	4.6

**Table 6:** Comparison results on the VisDrone-DET dataset using different downsampling methods inside TRD. The K denotes the size of the convolution block. We use YOLOv8-S as a baseline. Although larger convolutional kernels result in more substantial improvements in accuracy, this benefit comes at the expense of significantly increased computational costs. Therefore, the use of average pooling can better balance the detection accuracy and computational cost.

Method	AP (%)	AP_50_ (%)	AP_75_ (%)	AP_S_ (%)	AP_M_ (%)	AP_L_ (%)	#Param (M)	GFLOPs
SC (baseline)	23.6	40.0	21.8	12.7	33.4	42.0	11.2	14.4
TRD w/MaxPooling	24.7	40.8	25.1	14.6	37.2	45.7	12.0	16.3
TRD w/AveragePooling	24.9	41.0	25.5	14.8	37.5	45.6	12.0	16.3
TRD w/SC (K = 1)	24.9	41.0	25.4	14.9	37.4	45.7	12.4	16.8
TRD w/SC (K = 3)	25.5	42.0	26.0	**15.5**	38.3	46.1	15.2	21.0
**TRD w/SC (K = 5)**	**25.8**	**42.2**	**26.5**	**15.5**	**38.6**	**48.3**	20.8	29.4

## Data Availability

The VisDrone-DET dataset and the reference codes in this work are available at https://github.com/VisDrone/VisDrone-Dataset (accessed on 28 October 2019). The NWPU VHR-10 dataset used in this study is accessible from http://pan.baidu.com/s/1hqwzXeG (accessed on 24 November 2022).
